# Analysis of Global Research on Malaria and *Plasmodium vivax*

**DOI:** 10.3390/ijerph16111928

**Published:** 2019-05-31

**Authors:** José Antonio Garrido-Cardenas, José Cebrián-Carmona, Lilia González-Cerón, Francisco Manzano-Agugliaro, Concepción Mesa-Valle

**Affiliations:** 1 Department of Biology and Geology, University of Almeria, 04120 Almeria, Spain; jcardena@ual.es (J.A.G.-C.); pepe_cebri@hotmail.com (J.C.-C.); cmesa@ual.es (C.M.-V.); 2 Regional Center for Public Health Research, National Institute of Public Health, Tapachula, Chiapas 30700, Mexico; lgonzal@insp.mx; 3 Department of Engineering, University of Almeria, CeiA3, 04120 Almeria, Spain

**Keywords:** malaria, *Plasmodium vivax*, chloroquine, *Plasmodium falciparum*, Scopus

## Abstract

*Background*: Malaria is one of the infectious diseases of greatest interest to the scientific community and of greatest concern to international health authorities. Traditionally, the focus has been on *Plasmodium falciparum*, the parasite that causes the most severe form of the disease in Africa. However, in the last twenty years, the *Plasmodium vivax* parasite, responsible for a large number of cases in Latin America, the Middle East, South and Southeast Asia, the Horn of Africa, and Oceania, has also generated enormous interest due, among other things, to the published evidence that it can cause severe malaria. *Methods*: In this paper, the international scientific publication on malaria and *P. vivax* has been analyzed using the Scopus database to try to define global trends in this field of study. *Results*: It has been shown that events such as the emergence of resistance to certain drugs can break a trend. The important role of non-malaria-endemic countries such as the USA or Switzerland in malaria research is also evident. *Conclusions*: International cooperation will be essential for the eradication of the disease. Moreover, in this sense, the general vision given by the bibliometric analysis of malaria caused by *P. vivax* is fundamental to paint the picture regarding the current situation and encourage international cooperation and control efforts.

## 1. Introduction

Malaria is a disease that has affected the human population since ancient times. Today, it remains one of the infectious diseases with the highest morbidity and mortality rates, with 219 million estimated cases occurred worldwide in 2017, according to WHO, and half a million people dying each year worldwide, many of them children under five [1]. Because of this, a series of international programs have been initiated that aim to reduce and eradicate malaria, such as malERA, a research agenda for malaria elimination and eradication [2]. Human malaria can be caused by different species of the *Plasmodium* parasite. *P. falciparum* and *P. vivax* are the most important, with more than 95% of the cases diagnosed in the world, but there are others such as *P. malariae*, *P. knowlesi*, *P. ovale wallikeri*, and *P. ovale curtisi* [3].

*P. falciparum* is the most severe form of the disease in Africa, where more than 90 percent of all malaria cases occur. For this reason, this is the best-characterized species [4]. However, in recent years, the infections caused by *P. vivax* are increasing in significance because of evidence for severe malaria *P. vivax* infection [5]. In addition, *P. vivax* has recently received a large amount of attention, as it is the species with the largest geographical distribution, being reported fundamentally in Latin America, the Middle East, South and Southeast Asia, the Horn of Africa and Oceania [6]. This attention received by *P. vivax* is not only scientific, as illustrated by the increase in the number of published articles on this subject, but has also increased interest at the health level, trying to improve the diagnosis of malaria by these species or the specific treatments. The last factor that makes *P. vivax* a parasite of growing interest is its very hard elimination, with a high number of symptomatic relapses in malaria patients [7].

The malaria parasite is transmitted by a female *Anopheles* mosquito, inoculating sporozoites into the human host [8]. The sporozoites reach human liver cells, where they transform to give rise to another form called the merozoite. The merozoite reaches the erythrocytes, through the bloodstream, and multiples to produce new merozoites. Some of the merozoites released after the breakage of the erythrocytes are transformed into gametocytes. If the *Anopheles* mosquito bites an infected individual, then this is how the merozoites re-enter it. Inside the mosquito, in its midgut, sexual reproduction takes place, generating zygotes and developing into oocysts. These, as they grow and break, release sporozoites that invade the mosquito’s salivary glands. The parasite will then in the proper form to infect a new individual [9]. A fundamental difference in the life cycle of *P. vivax* is that it can successfully finish its development cycle in the mosquito at lower temperatures and faster than *P. falciparum*. 

The complexity of the parasite’s life cycle, its great genetic variability, and the numerous mechanisms it can develop to avoid the host’s immune response, make it very difficult to find a vaccine to suppress human malaria [10,11]. In addition, the biology of *P. vivax*, as opposed to P. falciparum, makes it harder to control and eliminate and is the main reason for the higher vectorial capacity of *P. vivax.* Other reasons are related to the presence of hypnozoites (latent hepatic forms), which lead to multiple relapses and low parasites densities, which in turn makes diagnosis difficult and delays treatments [12]. On the other hand, there are no suitable experimental models for the analysis of a hypothetical vaccine. Despite this, finding a vaccine to eradicate malaria caused by *P. vivax* has become a fundamental objective for the WHO and the scientific communities around the world. 

Bibliometric analyses provide fundamental scientific tools allowing for objective quantification of a scientific fact. These show the level of current knowledge in a scientific field by the compilation of data obtained from bibliographic databases. Bibliometrics facilitate comprehension and elaborates a real image of the research activity. In this study, the analysis of the international scientific publication on malaria and *P. vivax* has been raised to establish worldwide trends. It is essential for defining the research lines in malaria and creating synergies in order to achieve the WHO objective of eradicating malaria [13].

## 2. Materials and Methods

The evolution of the electronic age has led to the development of numerous scientific databases on the World Wide Web, which offer search facilities on a particular issue. Among them, some allow the opportunity to analyze citations from published works. The main scientific databases covering medical terms are PubMed, Scopus, Web of Science, and Google Scholar. PubMed focuses essentially on medicine and biomedical sciences, while Scopus, Web of Science, and Google Scholar cover most scientific fields. Usually, bibliometric studies are based on the keywords indexed by the published works. The main differences between these databases are PubMed (no limit), Scopus (30), Web of Science (15), and Google Scholar (no limit). However, in indexed journals and conference proceedings, there is usually a limit of about 6 keywords per published work so the limit of 15 is considered adequate for bibliometric works. However, PubMed’s search function, which is designed to search for medical documents, are exceptional and offer a service that other search engines do not. In this database, the search results can only be sorted by general characteristics such as publication date or author, and therefore, this is not very useful to get an overview of a topic [14]. Nowadays, mainly Web of Knowledge and Scopus allow large-scale downloads of bibliographic information from indexed publications and so most of the worldwide bibliometric works are based on one of these two databases. Scopus is the database that indexes the largest number of publications and conference proceedings when compared to the other three databases mentioned. Elsevier’s Scopus database is currently the largest abstract and citation database of peer-reviewed literature, and although another database, the Web of Science (WoS), is also available, it lists fewer titles whereas Scopus lists 84% of WoS titles compared to only 54% of Scopus tiles listed by WoS [15,16].

Scopus is usually considered the largest abstract and citation database of peer-reviewed scientific literature in the world. It contains more than 35,000 titles belonging to more than 10,000 publishers. For this reason, in order to analyze who, how, where, and what is being researched in a given scientific field, the most commonly used option is to use this database. Thus, it is common to find bibliometric works in many scientific fields using Scopus [17,18,19]. In short, Scopus is the most effective search engine and provides an overview of the subject. For extensive and in-depth research in the area of life sciences and closely related topics, PubMed should be considered as well [14]. For this reason, Scopus was the database of choice for this analysis.

**Figure 1:**
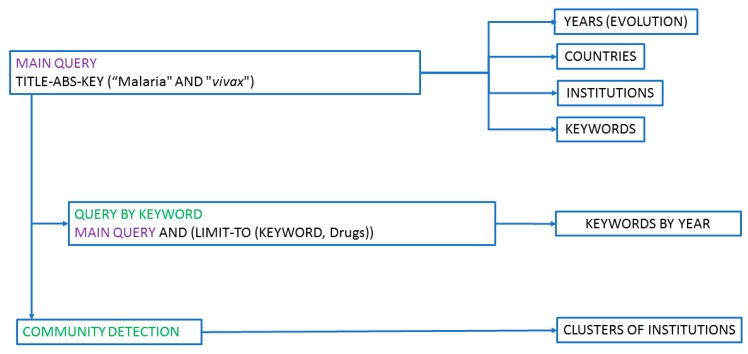
Methodology for searching the different analyses.

In this work, a full search of the Elsevier Scopus database was conducted using TITLE-ABS-KEY (malaria and *vivax*) as the search query. This resulted in 11,166 documents being obtained between 1916 and 2018, the last full year from Scopus database. It should be noted that varying the search criteria, or subsequent modifications of Scopus, can give significantly different results. That keywords entered by the author or publisher may not strictly conform to the subject matter of the articles should also be considered. Notwithstanding the previous, Scopus is considered a valid option for this type of analysis. In keyword analysis, terms with identical meanings were grouped together (e.g., *Plasmodium vivax* and *P. vivax*), and terms that do not contribute to this analysis were discarded (e.g., article). The aspects that have been studied are the progression in the number of publications per year, the distribution of publications by institutions and by country, and the keywords (Figure 1). For the detection of scientific communities, understood as the set of nodes connected to each other in a complex network, the software tool VOSviewer [20] (http://www.vosviewer.com/) was used. This software has been used to create graphs in which each institution is represented by a node and the connections between two nodes represent the collaboration between the two institutions represented. 

## 3. Results and Discussion

### 3.1. Progression of Scientific Output

**Figure 2:**
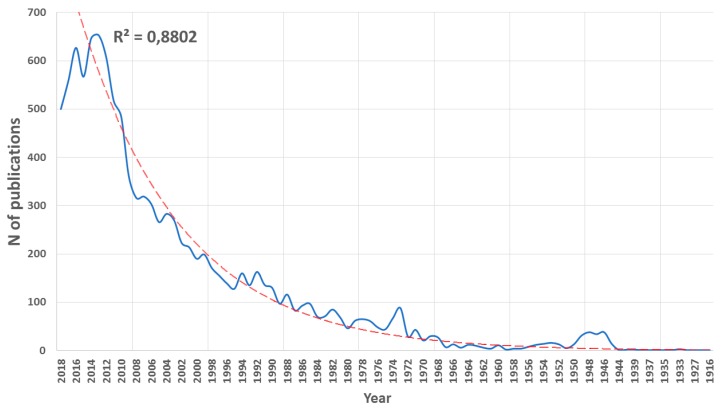
The trend of the number of publications in malaria and *Plasmodium vivax*, from the years 1916–2018.

The search returned 11,166 documents. Figure 2 shows the evolution trend of the number of documents on *Plasmodium vivax* and malaria since the first article was published. As shown in Figure 2, the first article published on this is dated in 1916 [21]. This early entry in the first article shows the strong interest that the scientific community has placed on malaria over the years.

The maximum number of annual publications was obtained in 2014, reaching a value of 646. The growth in the number of publications presents an exponential pattern, with an R^2^ coefficient close to 1. There are two years in which the trend is interrupted, and therefore, the R^2^ value is not even higher. These are the years 1946 and 1973. In these years, considering the trend line, the publications were higher than expected. The reason is that, on the one hand, in 1946, chloroquine was identified as a first-line blood schizontocide for *P. vivax* [22]. This led to an increased interest in malaria research and an increase in the publications. On the other hand, in 1973, there was intensification in the disease due to two reasons: the reduction in international aid programs from developed countries in the early 1970s, due to the lack of prospects for eradication; and the international economic crisis of 1973, which pushed up the price of insecticides. Between the years 2002–2010, the increase in the number of publications was three times greater than the whole previous period. This could be explained by the first malaria conference, Vivax Malaria Research: 2002 and Beyond, which took place in Bangkok, Thailand, 3–8 February 2002. This was a conference devoted entirely to *Plasmodium vivax* research and convened by the Multilateral Initiative on Malaria [23]. On the other hand, at the turn of the century, there was a general increase in funding for malaria control, and initiatives such as Roll Back Malaria, RBM, Partnership were born. This is a major worldwide platform for coordinated action towards a malaria-free world and is composed of international researchers, companies, and organizations.

### 3.2. Publication Distribution by Countries and Institutions

**Figure 3:**
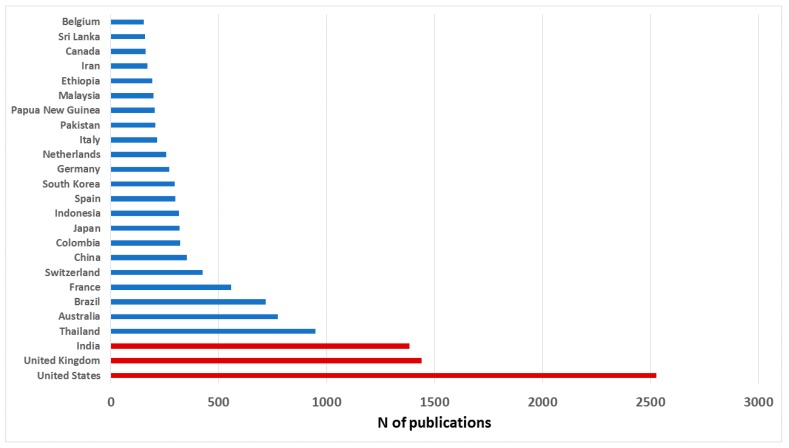
Representation of the countries with the highest number of publications on malaria and *P. vivax.*

**Figure 4:**
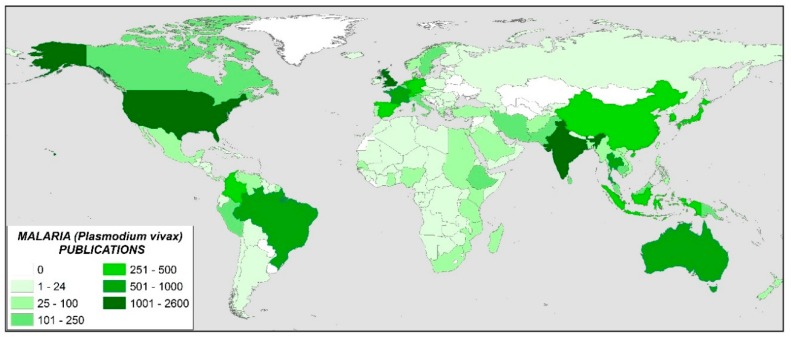
World map representing scientific publications by the intensity of color.

To get an overview of worldwide research on a specific subject, one of the aspects most considered is the study of publications by countries and institutions [24]. Note that when an article has several affiliations, the indexation implies that the article is attributed to each of them; therefore, the sum of articles by countries could be greater than the sum of articles obtained with the search term. Figure 3 shows the distribution by country of the scientific publication on malaria and *P. vivax*. In 159 countries, at least one article on *P. vivax* has been published, and 25 countries, with at least 150 publications, represent 75% of the total. Note that the same article may be signed by authors from different countries. Figure 4 shows the data on a world map with colors identifying the number of manuscripts that are published in each country. The United States of America, United Kingdom, and India, top the ranking with 2528, 1439, and 1383 publications, respectively. To normalize the results, the data have been referenced to the population of each country, based on the 2018 statistics obtained from the website [25] (Table 1). In this case, it can be observed that there are 10 countries that publish at least 7 articles per million inhabitants. These are (ranked from highest to lowest): Switzerland, Australia, Papua New Guinea, United Kingdom, Netherlands, Thailand, Belgium, France, United States, and Sri Lanka. 

In relative terms, Switzerland very high scientific output is striking. This can be explained by two reasons. First, Switzerland must be considered an international power in innovation. It is no coincidence that the development of an effective malaria vaccine is taking place in the country. The pharmaceutical industry is well established in this country, and there are numerous public and private research and development institutions around this type of industry [26]. On the other hand, Switzerland is home to international agencies and institutions concerned with global health care. The Swiss Agency for Development and Cooperation (SDC), an agency of the Swiss federal administration responsible for coordinating cooperation and humanitarian aid activities, and the World Health Organization, whose headquarters are in Geneva, stand out in this regard.

Finally, Table 1 also reflects the relative wealth of each country based on the value of GDP per capita (IMF data). Thus, it can be observed that the 10 countries studied above are divided into two groups. Seven high-income countries (Switzerland, Australia, United Kingdom, Netherlands, Belgium, France, and United States), with more than 40,000 GDP per inhabitant, and three low-income countries (Papua New Guinea, Thailand, and Sri Lanka), with less than 7000. The motivations in one or the other case are different. While in the former, the interest is purely scientific, for the latter it is a question of survival. In these three countries, malaria is an endemic disease, although in terms of survival, there is very little malaria in Thailand, and Sri Lanka has eliminated all malaria.

**Figure 5:**
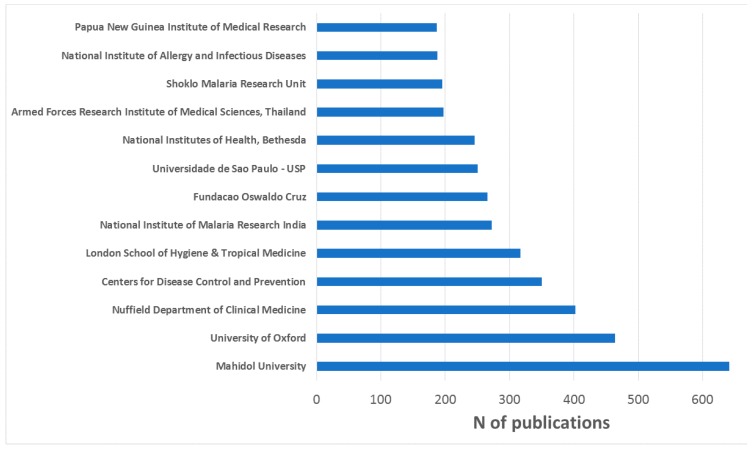
Main institutions in terms of scientific publication in malaria and *P. vivax.*

Figure 5 shows the 13 institutions with at least 180 publications on malaria and *P. vivax*. Of these, four are from USA (Centers for Disease Control and Prevention, National Institutes of Health in Bethesda, National Institute of Allergy and Infectious Diseases, and Armed Forces Research Institute of Medical Sciences known as AFRIMS), three are from the UK (the University of Oxford, Nuffield Department of Clinical Medicine, and London School of Hygiene and Tropical Medicine), two are from Thailand (Mahidol University and Shoklo Malaria Research Unit), two are Brazilian (Fundacao Oswaldo Cruz and Universidade de Sao Paulo), and one is Indian (National Institute of Malaria Research of India). Note that there may be several affiliations within the same institution but the database considers them separately respecting the decision of the authors. 

**Figure 6:**
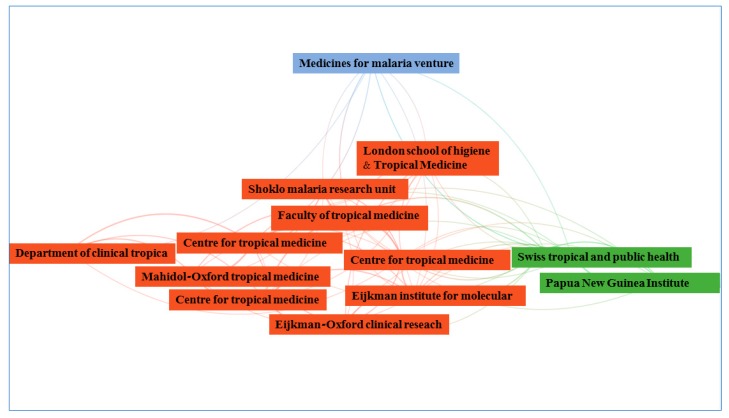
Distribution by communities of the main institutions.

As mentioned above, these five countries along with Australia, are the most relevant in scientific publications on this topic. In addition, it is surprising that among these, there is also an institution from Papua New Guinea. This is the Papua New Guinea Institute of Medical Research, also known as PNGIMR, that has the support of the World Health Organization (WHO). Figure 6 shows a distribution by communities of the main institutions. It can be observed that most institutions are grouped into a cluster whose central element is the faculty of tropical medicine of the Mahidol University (Thailand). Each line of union between the nodes represents the relationships established between the institutions. Thus, the relations are quite complex, and that they are observed not only between the different elements of this large cluster but also with the other institutions of the two additional minority clusters. The two smaller clusters are made up of the Papua New Guinea Institute of Medical Research and the Swiss Tropical and Public Health Institute, in one, and the Medicines for Malaria Venture (MMV) in the other.

### 3.3. Keyword Analysis

**Figure 7:**
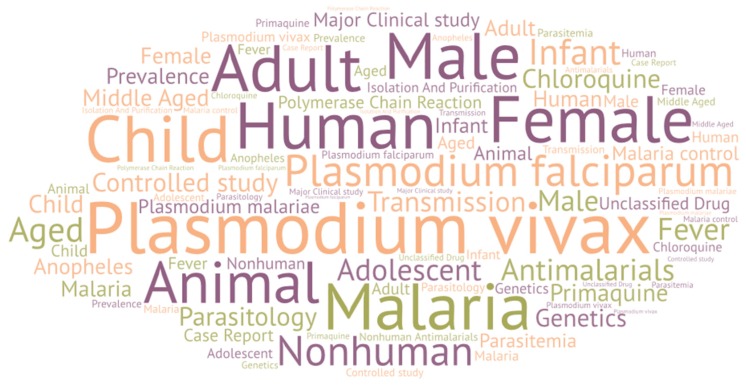
Word cloud with the main keywords.

In the analysis of the keywords, if one of them does not contribute anything to the study then it must be eliminated, e.g., “article.” In the second place, all terms that refer to the same concept must be grouped together, e.g., “*Plasmodium vivax*” and “*P. vivax*.” Figure 7 shows, using a word cloud, that the 32 keywords are in more than 1000 publications on malaria and *P. vivax*. In Figure 7, the relative size of each word is directly proportional to the number of times the keyword is present in the analyzed documents. As expected, *Plasmodium vivax*, human, and malaria, with 9826, 9428, and 8572, respectively, stand out.

**Figure 8:**
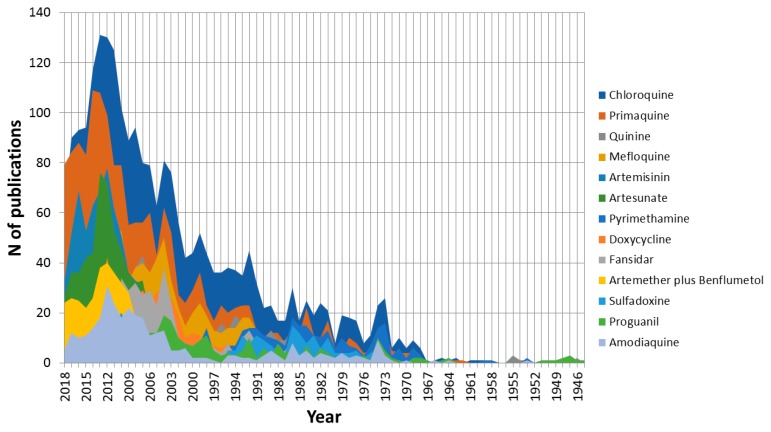
Time progression of antimalarial drugs in the fight against malaria caused by *P. vivax.*

Among the 32 keywords with more than 1000 presences in the analyzed articles are two drugs, chloroquine and primaquine, present in 2395 and 1682 documents, respectively. Furthermore, 11 other keywords related to drugs are among the 160 most used keywords. These are ranked in order of their importance: quinine, mefloquine, artemisinin, artesunate, pyrimethamine, doxycycline, fansidar (actually, the trade name for sulfadoxine/pyrimethamine), artemether plus benflumetol, sulfadoxine, proguanil, and amodiaquine. Figure 8 shows that since 1946, the evolution of malaria and *P. vivax* research in relation to the different drugs. As already mentioned, the most important drug as keyword is chloroquine. Although considering only the last 5 years, the relative importance of primaquine is similar. This is because the WHO indications are that, in areas where chloroquine maintains its efficacy, this must be the drug used against malaria caused by *P. vivax* [27]. On the other hand, primaquine has been shown to be highly effective in acting against hypnozoites, which are the predominant latent forms in *P. vivax*. Furthermore, until recently, it was the only approved hypnozoiticide. For these reasons, to avoid relapses caused by it, the administration of primaquine is appropriate although there are threats from incomplete compliance with standards and the development of tolerance, although the real problem in the supply of this drug is its potential toxicity due to the deficiency, in patients, of the enzyme G6PD [28]. Figure 8 also shows the relative importance of different drugs at any given time. For example, mefloquine had a relative maximum in 2004 and how, subsequently, it has been losing relevance due to its scarce further use. On the contrary, artemisinin—and its derivative artesunate—has a fundamental importance in recent years, being the most effective drug against all forms of multidrug-resistant *P. falciparum* [29]. Other drugs such as fansidar (sulfadoxine/pyrimethamine) or proguanil have practically no importance in recent publications on malaria and *P. vivax*, highlighting the trend in the use of these in the treatment of the disease.

Also, it can be seen that the five species of *Plasmodium* that have been shown to cause malaria in humans (*P. vivax*, *P. falciparum*, *P. malariae*, *P. ovale,* and *P. knowlesi*) are among the 100 most used keywords. Finally, five countries are also present among the most important keywords: India, Thailand, Brazil, China, and Papua New Guinea. These are countries where malaria caused by *P. vivax* is endemic and they are at the focus of many efforts by the international scientific community to eliminate malaria.

## 4. Conclusions

Malaria is still one of the world’s major health problems today; both for its extent and for the priority consideration it has received from public and private organizations concerned with human health. For this reason, malaria is a topic that is increasingly published in scientific journals with impact factors. Moreover, within this theme, malaria caused by *P. vivax* is currently receiving special interest. The present study has shown that the growth in the number of publications is exponentially curved, demonstrating the enormous interest that *P. vivax* causes in the international scientific community. This trend is only interrupted by two moments in history when interest in malaria has broken the norm. Thus, it becomes clear how bibliometric analysis of a given subject allows fundamental facts or moments to be identified. In this specific case, it is the discovery of a drug, chloroquine, which proved to be useful in the fight against malaria, and the rebound in the number of malaria cases in the early 1970s due to the relaxation of the alert level in the international scientific community. 

On the other hand, the most important countries in terms of scientific publication have been identified. In global terms, the USA, UK, and India stand out above the rest. However, when a more exhaustive analysis is carried out, and both the population and the wealth of the country are considered, it is observed that there are other countries of greater relative importance. Thus, Switzerland has been found to be the country that devotes the most relative effort to the fight against malaria. This is no coincidence. This country is a biotechnological benchmark and is home to many international public and private bodies that have been working to eliminate malaria for decades. The identification of countries such as Switzerland highlights the importance of combining technological, scientific, and political efforts of public and private initiatives in the fight against the disease. This union of efforts is evidenced by the study of the relationships between the most outstanding institutions and scientists in the field of malaria and *P. vivax*. In most cases, there are collaborations that dilute the borders between rich countries that are devoting efforts to fight malaria, such as the UK, and countries where the disease is a real public health problem, such as Thailand. 

Bibliometric studies not only give an overview of the current state of a scientific issue but can help to understand policy decisions and shape future scientific research. For this reason, an analysis of the keywords has been carried out allowing us to identify the main sectors in which the greatest efforts are being focused on research on malaria and *P. vivax*. Of these, studies on antimalarials stand out. The elements that define the lines of international economic investment and objectives in research projects are the progression of trends, the recommendations of the WHO, the updating of studies on the effectiveness of drugs, and the existence of resistance to them. It is important to have a general view of the subject in order to focus on the strategies that are still valid and to open up new promising lines of research.

## Figures and Tables

**Table 1:** Leading countries in terms of scientific research and publication in terms of population and wealth cited.

Country	Publications (N)	Population (P) (Mill. of Inhabitants)	N/P	GDP Per Capita
United States	2528	329,093	7682	62,332
UK	1439	66,959	21,491	41,951
India	1383	1,368,738	1010	1965
Thailand	948	69,306	13,678	6984
Australia	773	25,089	30,812	56,920
Brazil	718	212,393	3381	8988
France	559	65,481	8537	42,684
Switzerland	427	8608	49,605	82,365
China	353	1,420,062	249	9476
Colombia	321	49,850	6439	6580
Japan	318	126,855	2507	39,975
Indonesia	315	269,536	1169	3729
Spain	299	46,441	6438	30,942
South Korea	295	51,339	5746	32,256
Germany	270	82,439	3275	48,872
Netherlands	256	17,133	14,942	53,114
Italy	215	59,217	3631	35,226
Pakistan	206	204,596	1007	1486
Papua New Guinea	205	8587	23,873	3028
Malaysia	198	32,454	6101	11,247
Ethiopia	192	110,136	1743	781
Iran	171	82,821	2065	5059
Canada	162	37,280	4345	46,513
Sri Lanka	158	21,019	7517	4425
Belgium	153	11,563	13,232	48,603
